# Supplementary material for: Biological Evaluation of Photodynamic Effect Mediated by Nanoparticles with Embedded Porphyrin Photosensitizer
Source: Int J Mol Sci. 2022 Mar 25;23(7):3588. doi: 10.3390/ijms23073588 (PMC8998438; doi:10.3390/ijms23073588)
Supplement: Supplementary file 1 [file ijms-23-03588-s001.zip › ijms-1590207-supplementary.pdf]

SUPPORTING INFORMATION

Biological Evaluation of Photodynamic Effect Mediated by Nanoparticles with Embedded Porphyrin Photosensitizer

Ludmila Žárská[1], Zuzana Malá[1], Kateřina Langová[1], Lukáš Malina[1], SvatoplukBinder[1], Robert Bajgar[1, 2], Petr Henke [3], Jiří Mosinger [3], Hana Kolářová[1*]

[1]*Department of Medical Biophysics, Faculty of Medicine and Dentistry, Palacky University in Olomouc, Hnevotinska 3, 775 15 Olomouc, Czech Republic*

[2]*Institute of Molecular and Translation Medicine, Faculty of Medicine and Dentistry, Palacky University in Olomouc, Hnevotinska 5, 775 15 Olomouc, Czech Republic*

[3]*Department of Inorganic Chemistry, Faculty of Science, Charles University, Hlavova 2030, 12843 Prague 2, Czech Republic*

*** Corresponding Author*:*** *hana.kolarova@upol.cz*

Content

Figure S1: Normalized UV-Vis spectra of TPP-NP and TPP

Figure S2. Normalized fluorescence spectra of TPP-NP and TPP

Absorption spectra of TPP encapsulated in polystyrene matrix of nanoparticles are almost identical to the absorption spectrum of TPP in toluene. There is a small contribution of the protonated form of TPP (Protonated form has redshifted the Soret band from 420 to 440 nm) (Figure S1), which is better visible from emission spectra where a new band of the protonated form (680 nm) is clearly visible between two bands of the non-protonated form (650 and 717 nm) (Figure S2). UV-Vis absorption spectra were recorded on Varian 4000 spectrometer equipped with an integration sphere. Steady-state fluorescence spectra were monitored on an FLS 980 spectrofluorometer (Edinburgh Instruments).

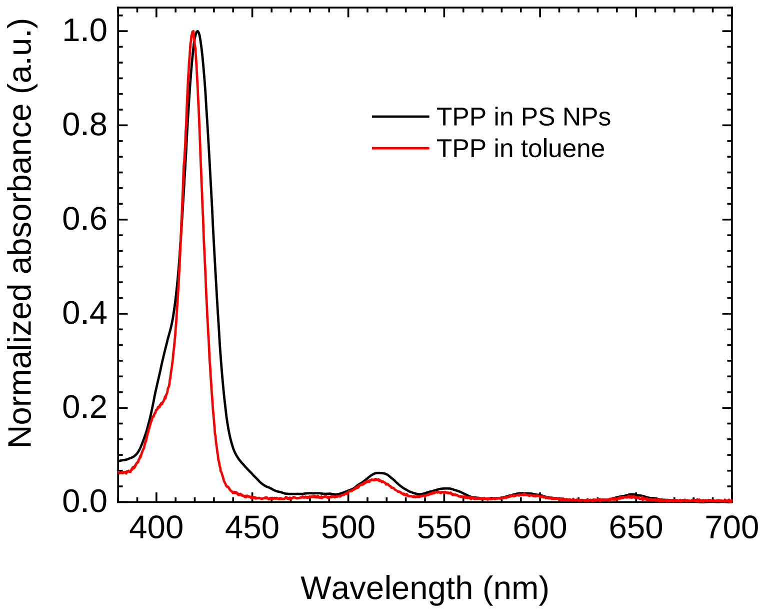

Figure S3: Normalized UV-Vis spectra of ***TPP-NPs*** *in water and TPP dissolved in toluene*

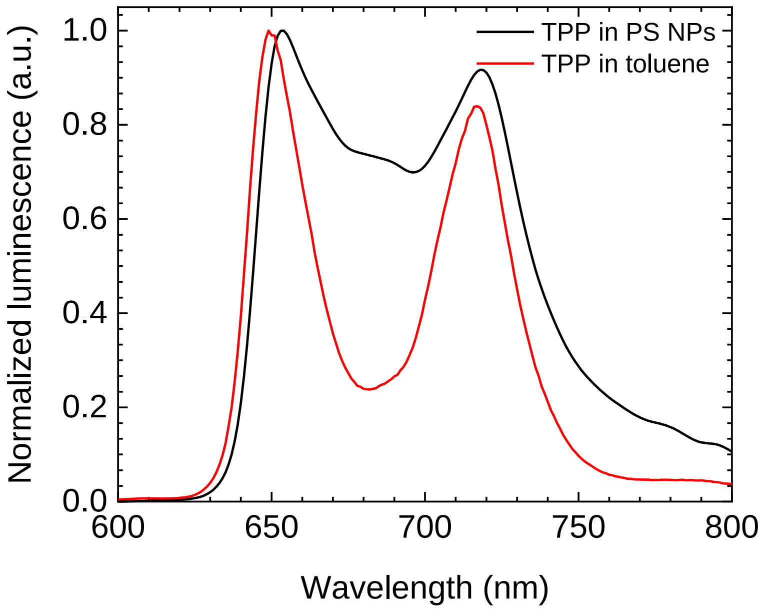

Figure S4. Normalized fluorescence spectra of TPP-NPs suspensions and TPP dissolved in toluene. Excitation at λ_{exc} = 516 nm.
